# Identification of monogenic diabetes in an Australian cohort using the Exeter maturity-onset diabetes of the young (MODY) probability calculator and next-generation sequencing gene panel testing

**DOI:** 10.1007/s00592-023-02193-x

**Published:** 2023-10-09

**Authors:** Sunita M. C. De Sousa, Kathy H. C. Wu, Kevin Colclough, Lesley Rawlings, Andrew Dubowsky, Melissa Monnik, Nicola Poplawski, Hamish S. Scott, Michael Horowitz, David J. Torpy

**Affiliations:** 1 Endocrine and Metabolic Unit, Royal Adelaide Hospital, Adelaide, Australia; 2 Adult Genetics Unit, Royal Adelaide Hospital, Adelaide, Australia; 3 Adelaide Medical School, University of Adelaide, Adelaide, Australia; 4 Clinical Genomics, St Vincent’s Hospital, Darlinghurst, NSW Australia; 5 School of Medicine, University of New South Wales, Sydney, NSW Australia; 6 Discipline of Genomic Medicine, Faculty of Medicine and Health, University of Sydney, Sydney, NSW Australia; 7 School of Medicine, University of Notre Dame, Sydney, NSW Australia; 8 Exeter Genomics Laboratory, Royal Devon University Healthcare NHS Foundation Trust, Exeter, UK; 9 Department of Genetics and Molecular Pathology, SA Pathology, Adelaide, Australia; 10 Centre for Cancer Biology, an alliance between SA Pathology, The University of South Australia, Adelaide, Australia

**Keywords:** Maturity-onset diabetes of the young, Monogenic diabetes, Mitochondrial diabetes, Next-generation sequencing, Genetic testing

## Abstract

**Aims:**

This study aims to describe the prevalence of monogenic diabetes in an Australian referral cohort, in relation to Exeter maturity-onset diabetes of the young (MODY) probability calculator (EMPC) scores and next-generation sequencing with updated testing where relevant.

**Methods:**

State-wide 5-year retrospective cohort study of individuals referred for monogenic diabetes genetic testing.

**Results:**

After excluding individuals who had cascade testing for a familial variant (21) or declined research involvement (1), the final cohort comprised 40 probands. Incorporating updated testing, the final genetic result was positive (likely pathogenic/pathogenic variant) in 11/40 (27.5%), uncertain (variant of uncertain significance) in 8/40 (20%) and negative in 21/40 (52.5%) participants. Causative variants were found in *GCK*, *HNF1A, MT-TL1* and *HNF4A*. Variants of uncertain significance included a novel multi-exonic *GCK* duplication. Amongst participants with EMPC scores ≥ 25%, a causative variant was identified in 37%. Cascade testing was positive in 9/10 tested relatives with diabetes and 0/6 tested relatives with no history of diabetes.

**Conclusions:**

Contemporary genetic testing produces a high yield of positive results in individuals with clinically suspected monogenic diabetes and their relatives with diabetes, highlighting the value of genetic testing for this condition. An EMPC score cutoff of ≥ 25% correctly yielded a positive predictive value of ≥ 25% in this multiethnic demographic. This is the first Australian study to describe EMPC scores in the Australian clinic setting, albeit a biased referral cohort. Larger studies may help characterise EMPC performance between ethnic subsets, noting differences in the expected probability of monogenic diabetes relative to type 2 diabetes.

## Introduction

Monogenic diabetes refers to diabetes mellitus attributable to pathogenic variants in a single gene. Maturity-onset diabetes of the young (MODY) is the most common type of monogenic diabetes, accounting for ~ 2–5% of individuals with diabetes and characterised by an early onset, autosomal dominant inheritance, and insulin independence [1]. Other types of monogenic diabetes include neonatal diabetes (NDM) and mitochondrial diabetes. The predominant MODY subtypes are as follows: *GCK*-MODY, resulting in a high glycaemia set-point that typically should not be treated as it is not associated with microvascular complications of diabetes, and *HNF1A*- and *HNF4A*-MODY, which reduce glucose-mediated insulin secretion leading to progressive hyperglycaemia and complications, but with exquisite sulphonylurea sensitivity that often obviates the need for insulin [2]. Accordingly, diagnosing monogenic diabetes has important treatment implications, and it also informs prognosis and risk to family members [1]. Nonetheless, > 80% of MODY is undiagnosed or misdiagnosed as type 1 or 2 diabetes [3, 4].

Contemporary genetic testing for monogenic diabetes typically involves next-generation sequencing (NGS) to interrogate multiple implicated genes simultaneously. When the Exeter Genomics Laboratory transitioned from phenotype-guided single- or staged-gene sequencing to a 29-gene NGS panel, mutations were detected in a further 15% of individuals with MODY and 18% with NDM [5].

Drawing on data from 1191 participants, Shields et al. formulated the 8-item Exeter MODY probability calculator (EMPC, www.diabetesgenes.org) to guide which individuals benefit most from genetic testing. The EMPC generates a pre-test probability of testing positive by MODY genetic testing (i.e. a positive predictive value, PPV) that was shown to be more accurate than previous categorical clinical criteria. The authors recommended proceeding to genetic testing at cutoffs of > 10% for individuals treated with insulin within 6 months of diagnosis and > 25% for individuals who are not treated with insulin within 6 months of diagnosis [1]. Importantly, this study only included white Europeans. EMPC performance is expected to vary with ethnicity of the patient population, not because of the rate of monogenic diabetes which is expected to be stochastic (apart from uncommon founder mutations), but because of the differing rates of type 1 and type 2 diabetes relating to polygenic factors. Another limitation of the EMPC is that the original study looked only at *GCK*, *HNF1A* and *HNF4A* variants. However, the overlapping presentation between MODY subtypes and with other monogenic diabetes types including mitochondrial diabetes [6] suggests that the EMPC may guide broader monogenic diabetes genetic testing, particularly when simultaneous gene testing is available through NGS.

Seven studies have assessed the utility of the EMPC against genetic testing outcomes (Table 1). As most participants have been preselected based on other criteria suggestive of monogenic diabetes, these enriched populations are expected to have a higher MODY prevalence compared to unselected populations. Notwithstanding this limitation, these studies show that the observed PPV for a given EMPC cutoff varies between countries, highlighting the need for EMPC data from the specific multiethnic demographic of the Australian clinical setting. The single Australian study performed thus far described use of the EMPC with a 22-gene panel test performed in 25 participants with EMPC scores > 25% and variants identified in 0/13 non-Europeans versus 7/12 Europeans [7]. As participants in this study were recruited by various means including database searches and advertising rather than routine care, real-world Australian data are still required.

**Table 1:** Studies reporting on genetic testing yield with use of the Exeter MODY probability calculator (EMPC)

Study	Country	Participants	Monogenic diabetes type(s) assessed	Outcome
Thomas [19]	UK	36 adult probands (four non-White Europeans) referred for monogenic diabetes testing	MODY and mitochondrial DM	20% EMPC cutoff → 50% PPV
Ang [20]	Singapore	71 multiethnic Asian adults (59 Chinese, 14 Malay and 11 Indian) preselected on features, ( e.g., pancreatic autoantibody status) to exclude obvious T1DM and T2DM	MODY, monogenic insulin resistance and lipodystrophy	62% EMPC cutoff → 30% PPV
Haliloglu [21]	Turkey	54 Turkish children preselected by criteria including pancreatic autoantibody status	MODY	75% EMPC cutoff + additional features, e.g. HbA1c → 57% PPV
Davis [7]	Australia	25 Australians of mixed ethnicities recruited by various means, e.g., advertising	MODY, NDM	25% EMPC cutoff → 28% PPV
Tarantino [22]	Brazil	34 Brazilian probands with previous clinical diagnosis of monogenic diabetes	*GCK*-MODY and *HNF1A*-MODY	50% EMPC cutoff → 48% PPV; 75% EMPC cutoff → 53% PPV
Lee [23]	Korea	40 Korean probands preselected by criteria including pancreatic autoantibody status	MODY, NDM and lipodystrophy	50% EMPC cutoff → 15% PPV
Da Silva Santos [24]	Portugal	73 Portuguese probands preselected by criteria including pancreatic autoantibody status	All monogenic diabetes	25% EMPC cutoff → 65% PPV; newly derived 36% EMPC cutoff → 74% PPV

*PPV* positive predictive value (i.e. mutation detection rate), *T*1*DM* type 1 diabetes mellitus and *T*2*DM* type 2 diabetes mellitus

Better identifying who warrants monogenic diabetes genetic testing is valuable given the treatment implications, and a timely consideration now that genetic testing costs are falling with use of more economical NGS over Sanger sequencing [8]. Achieving a molecular diagnosis of MODY may be increasingly important with the influx of new hypoglycaemic agents, meaning that affected individuals may be less likely to chance upon a spectacular sulphonylurea response which has traditionally been a clue to MODY.

We undertook a state-wide 5-year retrospective cohort study of adults undergoing monogenic diabetes genetic testing to describe the prevalence of monogenic diabetes in an Australian referral cohort, in relation to Exeter maturity-onset diabetes of the young (MODY) probability calculator (EMPC) scores and next-generation sequencing with updated testing where relevant.

## Methods

We reviewed the South Australian Adult Genetics Unit database to identify all adults who underwent genetic testing for monogenic diabetes in 2017–2021. In our state, the typical pathway of monogenic diabetes genetic testing is for patients to be referred to the South Australian Adult Genetics Unit by their treating clinician based on overall clinical suspicion rather than any specific probability tool. Individuals undergoing cascade testing for a known familial variant were excluded.

Apart from one participant who had upfront testing for mitochondrial diabetes, all participants underwent NGS using germline DNA extracted from peripheral blood, and the Illumina NextSeq Sequencing System with either an Agilent, Roche or IDT capture depending on the testing laboratory. Either all genes (exome sequencing platform) or selected genes (custom sequencing panels) were sequenced. Analysis was only performed for monogenic diabetes genes. Copy number variants were interrogated by NGS analysis and/or dedicated multiplex ligation probe amplification (MLPA) using MRC-Holland Salsa MLPA kits. Mitochondrial DNA testing for the most common cause of mitochondrial diabetes, NC_012920.1(MT-TL1):m.3243A>G, was performed during initial genetic testing in 10 participants. As this was a real-world audit of clinical practice utilising different laboratories over a 5-year period, there was variation in which genes were tested, which MLPA kits were employed and the degree of heteroplasmy detection in mitochondrial testing. Variants were classified according to the American College of Medical Genetics and Genomics classification system: benign, likely benign, variant of uncertain significance (VUS); likely pathogenic (LP) and pathogenic (P) [9]. Results were considered positive if an LP or P variant was found.

Updated genetic testing or re-analysis was performed in select individuals if clinically indicated at the time of the audit. This included mitochondrial DNA testing for m.3243A>G by a TaqMan genotyping assay using DNA from peripheral blood, with a 2% lower limit of heteroplasmy detection. Mitochondrial DNA testing was performed in participants with a maternal history of diabetes, negative or VUS results on initial monogenic diabetes genetic testing and absence of previous mitochondrial genetic testing.

Cascade testing for LP and P variants was performed in blood relatives who wished to be tested, using germline DNA and Sanger sequencing of the region containing the relevant variant.

The probability of finding a monogenic diabetes gene mutation was retrospectively determined in participants who had undergone genetic testing through use of the EMPC, with input of diagnosis age, sex, pharmacological requirement, time to insulin, current body mass index (BMI), current HbA1c, current age and parental history of diabetes. If the original referral contained an EMPC value, this was compared against the EMPC calculated for the purposes of this study. EMPC was not performed if diabetes diagnosis age was > 35 years as it has not been validated beyond this age [1]. Amongst eligible participants, we compared an EMPC cutoff of 25% against mutation detection rates. This EMPC cutoff was selected based on local practice and to reflect the original EMPC study by Shields et al*.* [1] and the single previous Australian study on this topic [7]. As the EMPC score represents a PPV, finding an observed PPV (i.e. mutation detection rate) that is comparable or better than the selected EMPC cutoff was considered supportive of EMPC utility.

Clinical and genetic test characteristics and EMPC performance parameters were assessed. Statistical analysis was performed using IBM SPSS Statistics 29.0 for between-group comparisons (Chi-squared/Fisher’s exact test for categorical variables, *t*-test/Mann–Whitney test for continuous variables) and EMPC receiver operator characteristic (ROC) curve analysis.

The study was approved by the Central Adelaide Local Health Network Human Research Ethics Committee (2021/HRE00232). All participants gave written consent to genetic testing. Due to the audit nature of the study, consent to publication was waived by the approving Ethics Committee in accordance with the National Health and Medical Research Council research guidelines.

## Results

### Study cohort

We identified 62 individuals who underwent monogenic diabetes genetic testing in a 5-year period between 2017 and 2021. After excluding 21 individuals who had cascade testing for a known familial variant and one individual who previously declined research involvement, the final cohort consisted of 40 participants (Table 2). Ethnicity was white European in 31/40 (77.5%), non-white European in 8/40 (20%) and unknown in 1/40 (2.5%) participants.

**Table 2:** Clinical and genetic test characteristics of the study cohort

	Overall cohort
White European ethnicity, %	78
Female, %	65
Age at initial diabetes diagnosis, year	28 (23–32)
Age at time of genetic test, year	36 (31–46)
Time from diabetes diagnosis to genetic test, year	6 (2–15)
BMI at time of genetic test, kg/m^b^	25 (21–28)
C-peptide level, pmol/L^a,b,c^	742 (541–1179)
High C-peptide (above upper limit of normal), %^a^	16
Low C-peptide (below lower limit of normal), %^a^	16
Negative pancreatic autoantibodies, %^d^	94
HbA1c at time of genetic test, %^e^	6.7 (6.0–7.2)
Diabetes in a parent, %	83
EMPC score, %^f^	46 (15–76)
Number of nuclear genes tested	34 (14–65)

Data are presented as medians and interquartile ranges (Q1–Q3) or proportions (%)

*BMI* body mass index, *EMPC* Exeter MODY probability calculator

^a^Amongst participants with a C-peptide result (*n* = 31)

^b^Latest level used

^c^Absolute level shown, slight variation in reference intervals between laboratories

^d^Proportion amongst participants with available results

^e^IFCC units, mmol/mol: 50 (42–55)

^f^Using results from time of genetic testing, median amongst participants in whom the EMPC could be calculated (*n* = 31)

Initial genetic test results showed VUS in 10/40 (25%), LP variants in 3/40 (7.5%) and P variants in 7/40 (17.5%) participants, with the remaining 20/40 (50%) participants having negative results. Thus, the initial result was positive (LP/P variant) in 10/40 (25%) participants. Missense, nonsense, frameshift and splicing variants were observed, in addition to a novel intragenic *GCK* duplication of exons 3–6 that was identified by NGS and confirmed by MLPA, although it remained a VUS. The 10 cases deemed positive by initial genetic testing included two participants with the m.3243A>G variant.

### Updated genetic test results

All 11 participants subsequently assessed for the m.3243A>G variant in the course of updated testing had a negative result.

Formal variant reviews were conducted in two cases with a VUS where it appeared amenable to variant reclassification. This resulted in a missense *GCK* VUS (NM_000162.3, c.554T>C (p.Leu185Pro)) being upgraded to LP, and a missense *PDX1* VUS (NM_000209.3, c.725C>T (p.Pro242Leu)) being downgraded to likely benign. The participant with the upgraded *GCK* variant was already clinically suspected to have *GCK*-MODY and had thus ceased pharmacological treatment. Identifying the causative variant allowed for cascade testing, which was positive in the single relative with diabetes (labelled as type 2 diabetes) and negative in three relatives without diabetes.

Using these updated genetic test results, the final classification of genetic variants was VUS in 8/40 (20%), LP in 4/40 (10%) and P in 7/40 (17.5%) participants (Table 3), with the remaining 21/40 (52.5%) participants having negative results. Thus, the final genetic result was positive in 11/40 (27.5%) participants. According to ethnicity, a causative variant was identified in 10/31 (32.3%) white Europeans versus 1/8 (12.5%) non-white Europeans (not significant,* P* = 0.4 using two-tailed Fisher’s exact test).

**Table 3:** Genetic and clinical features of participants with likely pathogenic or pathogenic variants

Gene	Variant class	Coding DNA change	Protein change	Sex	Ethnicity	Age at diabetes diagnosis, year	Current age, year	Taking insulin	Other conditions	Diabetes in a parent	Current BMI, kg/m^2^	Current HbA1c, % (IFCC, mmol/mol)	C-peptide, pmol/L	T1DM Abs	EMPC score, %
*GCK*	P	574C>T	Arg192Trp^a^	F	Caucasian	20	22	No		Yes	20	6.1 (43)	321	Pos	76
*GCK*	P	1133C >A	Ala378Asp	F	Caucasian	29	42	No	Subclinical hypothyroidism	Yes	28	6.6 (49)	536	Neg	36
*GCK*	P	574C>T	Arg192Trp^a^	M	Caucasian	26	33	No		Yes	23	6.0 (42)	nd	Neg	76
*GCK*	LP	952G>A	Gly318Arg	F	Caucasian	27	27	Yes		Yes	23	6.0 (42)	nd	Neg	49
*GCK*	VUS (→LP)	554 T>C	Leu185Pro	M	Caucasian	35	38	No		Yes	24	6.8 (51)	724	Neg	15
*HNF1A*	P	1522G>T	Glu508*	F	Caucasian	28	30	Yes		Yes	40	6.3 (45)	nd	nd	15
*HNF1A*	P	872dupC	Gly292Argfs*25	M	Caucasian	23	25	No	Dyslipidaemia	Yes	20	5.8 (40)	400	Neg	76
*HNF1A*	LP	404delA	Asp135Valfs*20	F	Caucasian	24	44	Yes	Dyslipidaemia	Yes	21	5.9 (41)	516	Neg	49
*HNF4A*	LP	406C>T	Arg136Trp	F	Caucasian	32	56	No		Unk	19	6.5 (48)	2367	Neg	n/a
*MT-TL1*	P	m.3243A>G	n/a	F	Caucasian	44	58	Yes	Deafness, short stature, myalgia and congenital single kidney	Yes	18	6.9 (52)	nd	Neg	n/a
*MT-TL1*	P	m.3243A>G	n/a	F	Indian	28	31	Yes	Miscarriage	Yes	20	6.0 (42)	1041	Neg	49

Current refers to time of genetic testing

*Abs* antibodies, *EMPC* Exeter MODY probability calculator, *F* female, *LP* likely pathogenic, *M* male, *n/a* not applicable, *nd* not done, *neg* negative, *P* pathogenic, *pos* positive, *T*1*DM* type 1 diabetes mellitus, *unk* unknown and *VUS* variant of uncertain significance

^a^No known relationship between the two affected individuals with this variant despite its rarity

Across all participants with a positive genetic result, cascade testing was positive for the relevant variant in 9/10 tested relatives with a history of diabetes and 0/6 tested relatives with no history of diabetes. The single relative with a history of diabetes but with a negative result on cascade testing demonstrated recent normoglycaemia with only an historical record of gestational diabetes, hence representing a phenocopy.

### EMPC performance

EMPC performance was assessed using final genetic status amongst the 31/40 (77.5%) participants in whom EMPC could be calculated. In seven participants, EMPC could not be calculated as age of diabetes diagnosis was > 35 years, and, in one participant each, BMI or parental status was unavailable. Genetic testing was positive in 2/9 (22.2%) participants in whom EMPC could not be calculated.

Median EMPC score was 49% in participants with a positive versus 34% in those with a negative, final genetic status. Amongst participants in whom EMPC could be calculated, EMPC score was ≥ 25% in 16/24 (66.7%) white Europeans and 3/6 (50%) non-white Europeans; an additional participant of unknown ethnicity had an EMPC score < 25%. Amongst all participants with EMPC scores ≥ 25%, a causative variant was identified in 37%. As the true PPV (37%) exceeded the defined EMPC cutoff (25%), this was considered supportive of EMPC utility in the Australian multiethnic setting. Performance characteristics of the other EMPC cutoffs discussed in the original EMPC study [1] are outlined in Table 4. A ROC curve analysis of EMPC scores did not reach statistical significance (AUC 0.619, 95% CI 0.414–0.824).

**Table 4:** Performance of the Exeter MODY probability calculator according to final genetic status

	EMPC ≥ 10% cutoff (%)	EMPC ≥ 25% cutoff (%)	EMPC ≥ 40% cutoff (%)	EMPC ≥ 60% cutoff (%)
Sensitivity	100	78	67	33
Specificity	18	45	55	68
PPV	33	37	38	30
NPV	100	83	80	71

Positive cases were those with likely pathogenic/pathogenic variants whereas all others were deemed negative for the purpose of assessing EMPC performance

*EMPC* Exeter MODY probability calculator, *NPV* negative predictive value and *PPV* positive predictive value

We looked at participants with previously recorded EMPC scores, noting that EMPC was not required for referral. We found that EMPC differed by ≥ 5% in 6/16 (37.5%) participants when comparing values calculated for this study versus values originally recorded by the referring/testing clinician. Maximum EMPC difference was 43%. At least in one participant, a large EMPC difference (42%) was driven by a large HbA1c decline from referral for genetic testing to time of genetic testing.

## Discussion

This is one of the largest published cohorts of Australian individuals with suspected MODY, and the first Australian study to assess EMPC scores in the clinic setting, albeit a biased referral cohort. We have shown that contemporary genetic testing with NGS gene panel testing, and updated testing where relevant, produces a high yield of positive results in individuals with clinically suspected monogenic diabetes and their relatives with diabetes. These results highlight the utility both of genetic testing in individuals with clinically suspected monogenic diabetes and of cascade testing in relatives, as well as the potential value of revisiting genetic testing in individuals with initially negative or ambiguous results. We have also shown that an EMPC score cutoff of ≥ 25% correctly yielded a positive predictive value of ≥ 25%, despite non-white Europeans comprising 20% of participants. This supports EMPC use in the particular multiethnic demographic of Australia, which should help identify individuals who will benefit from monogenic diabetes genetic testing as it becomes more affordable and accessible.

All but one participant in our study underwent genetic testing using NGS, with examination of up to 83 genes. Despite a median of 34 genes interrogated per participant, only four (*GCK*, *HNF1A*, *MT-TL1* and *HNF4A*) exhibited causative variants. An additional six genes exhibited VUS only. Another Australian study involving a 13-gene NGS panel in a cohort of children with diabetes similarly found that six genes accounted for all causative variants [10]. Nonetheless, new monogenic diabetes genes continue to be discovered, and exome sequencing backbones, as used in our local practice, facilitate reanalysis for emerging genes without the need for resequencing as traditionally required [11]. NGS is also superior to traditional Sanger sequencing because of its capacity to identify both single-nucleotide and copy number variants. In our study, one of the *GCK* VUS was a novel, multi-exonic intragenic duplication that is likely to be causative, and which would have been missed by Sanger sequencing. This is the first reported case of a duplication involving *GCK*; however, we have been unable to confirm pathogenicity of this variant.

Fewer non-white Europeans had a high EMPC than white Europeans, and they were less likely to have a positive genetic result, although these differences were not statistically significant. Larger studies are required to examine the differential utility of the EMPC in white European versus non-white European populations. The previous smaller Australian studies have shown that non-white Europeans are overrepresented amongst participants with negative results on MODY genetic testing [7, 12]. A UK study similarly found lower mutation detection in South Asians compared to white Europeans with suspected MODY (13% vs. 29%, *p* < 0.001) [13]. Lower mutation detection rates in non-Europeans likely relate to a higher background prevalence of T2DM. Asians with apparent young-onset T2DM often have normal BMI, at least partly due to visceral fat deposition producing insulin resistance, which may lead to an overestimation of MODY risk by the EMPC [14]. Alternatively, lower mutation rates in non-Europeans could reflect more false-negatives on genetic testing due to gene lists being derived from predominantly European cohorts. There is evidence that the variant landscape differs in Asian and Middle Eastern populations with suspected monogenic diabetes compared to historical literature [15, 16]. Variant pathogenicity may also be more difficult to determine in non-white Europeans, as attested to by reclassification of a *KCNJ11* VUS as a pathogenic variant in a Eurasian participant of the aforementioned Australian study by Davis et al*.* 2 years after the original publication [7, 17].

An EMPC cutoff of 25% was selected for examination in this study based on local practice and in line with the original EMPC study by Shields et al*.* [1] and the single other Australian study on this topic [7]. Shields et al*.* proposed a cutoff of 25% specifically for individuals not requiring insulin within 6 months of diagnosis, whereas a lower cutoff of 10% was suggested if insulin was started within 6 months. Distinct from these cutoffs, their data in fact supported higher EMPC cutoffs of 40% and 60% for maximum diagnostic accuracy in individuals with a label of type 1 and type 2 diabetes, respectively. However, these higher cutoffs yielded a lower sensitivity compared to 10% and 25% cutoffs. In real-world practice, whether the potential diagnosis of MODY is being compared against type 1 versus type 2 diabetes is often blurred, complicating a split model of screening. In our cohort, the higher EMPC cutoffs of 40% and 60% yielded PPVs that fell below these thresholds (Table 4). This highlights the importance of determining the optimal EMPC cutoff in a given population. Such data do not exist in Australia. We attempted to identify the optimal EMPC cutoff via ROC curve analysis, but the results were not significant within the limits of this small cohort.

Other considerations when applying the EMPC in clinical practice are the requirement for the age of diabetes diagnosis to be ≤ 35 years, which precluded EMPC use in 7/40 (17.5%) participants, and use of the latest HbA1c and BMI rather than baseline values, which appeared to contribute to the ≥ 5% EMPC discrepancy seen in 37.5% of our cohort. One person with a 42% EMPC discrepancy yielded a positive genetic result which would have been missed if a strict threshold for genetic referral/testing was mandated, as the referring EMPC score was just 7% using earlier HbA1c values compared to 49% using a much lower HbA1c that was achieved after insulin titration in this participant.

The mitochondrial variant m.3243A>G was identified in 2/40 (5%) of participants. One affected individual developed diabetes at 44 years and had short stature, bilateral deafness and longstanding myalgia. The other affected individual developed diabetes at 28 years without mitochondrial features. This supports recent literature calling for syndromic monogenic diabetes genes to be tested in all suspected MODY cases despite a lack of syndromic features [6, 18].

Study limitations include the small size of the cohort and selection bias. This was a convenience sample of individuals who were suspected to have monogenic diabetes by their treating physicians based on ad hoc clinical criteria and hence referred to the clinical genetics service for consideration of testing. The use of a referral cohort in this and other studies described in Table 1 means that the derived MODY prevalence rates in these enriched populations will be expectedly higher compared to when the EMPC is used in the general population. Future research could overcome the inherent bias of this and other referral cohort studies by performing both the EMPC and contemporary genetic testing across an entire population cohort of individuals with diabetes; the cost of this would be a major barrier, although MODY genetic testing was successfully undertaken in an unselected population-based paediatric diabetes cohort of 821 children led by Duncan et al*.* [10]. Another limitation of our study is that it was conducted in South Australia only, and these South Australian data do not necessarily reflect how the EMPC would perform nationally. There was also heterogeneity in genetic testing methodology, reflecting real-world practice with variations between testing clinicians and over time.

In conclusion, we have shown a high yield of positive genetic test results in individuals with clinically suspected monogenic diabetes and their relatives with diabetes using NGS, which enables simultaneously testing of multiple genes and identification of copy number variants. We have also outlined the value and nuances of the EMPC in a multiethnic Australian clinical setting, with an EMPC cutoff of 25% yielding a PPV of 37%. The EMPC is currently used by Australians in an ad hoc manner; more data are required before its use can be routinely recommended. We are now conducting a prospective study employing the EMPC in unselected individuals with diabetes. In addition, a national guideline on genetic testing in adults with suspected monogenic diabetes is currently underway. Greater familiarity and use of the EMPC as a screening tool together with the increasing accessibility of comprehensive genetic testing via NGS will help address the frequent misdiagnosis of monogenic diabetes as type 1 or type 2 diabetes and facilitate genotype-driven diabetes management.
